# Supplementary material for: Content-rich biological network constructed by mining PubMed abstracts
Source: BMC Bioinformatics. 2004 Oct 8;5:147. doi: 10.1186/1471-2105-5-147 (PMC528731; doi:10.1186/1471-2105-5-147)
Supplement: Additional File 5 — The original Chilibot query results of the term "long-term potentiation (LTP)" and 22 other terms, limiting the latest references analyzed to the years 1990, 1995, 2000, and 2004. [file 1471-2105-5-147-S5.bz2 › chilibotAdditionalFile5/ltp2000/html/SYNAPSIN I_TRKB.html]

 


 **SYNAPSIN I** and **TRKB** 
  
Found 1 abstracts in PubMed,  **1 abstracts were retrieved and analyzed**.  


---

 Search Google  |
 PDF files only 
|  EDU domain only 

---

- Nat Neurosci, 2000   **Synapsins as mediators of BDNF enhanced neurotransmitter release.** .
  We examined enhancement of synaptic transmission by neurotrophins at the presynaptic level.
  In a synaptosomal preparation, brain derived neurotrophic factor BDNF increased mitogen activated protein MAP kinase dependent **synapsin I** phosphorylation and acutely facilitated evoked glutamate release.
  PD98059, used to inhibit MAP kinase activity, markedly decreased **synapsin I** phosphorylation and concomitantly reduced neurotransmitter release.
  The stimulation of glutamate release by BDNF was strongly attenuated in mice lacking **synapsin I** and or synapsin II.
  These results indicate a causal link of synapsin phosphorylation via BDNF, **TrkB** receptors and MAP kinase with downstream facilitation of neurotransmitter release.
